# The effects of non-self-sustained oscillators on the en-trainment ability of the suprachiasmatic nucleus

**DOI:** 10.1038/srep37661

**Published:** 2016-11-21

**Authors:** Changgui Gu, Ming Tang, Jos H. T. Rohling, Huijie Yang

**Affiliations:** 1 Business School, University of Shanghai for Science and Technology, Shanghai 200093, China; 2 School of Information Science and Technology, East China Normal University, Shanghai 200241, P.R. China; 3 Big data research center, University of Electronic Science and Technology of China, Chengdu 610054, China; 4 Department of Molecular Cell Biology, Laboratory for Neurophysiology, Leiden University Medical Center, Leiden, 2300 RC, The Netherlands

## Abstract

In mammals, the circadian rhythms of behavioral and physiological activities are regulated by an endogenous clock located in the suprachiasmatic nucleus (SCN). The SCN is composed of ~20,000 neurons, of which some are capable of self-sustained oscillations, while the others do not oscillate in a self-sustainable manner, but show arrhythmic patterns or damped oscillations. Thus far, the effects of these non-self-sustained oscillatory neurons are not fully explored. Here, we examined how the proportion of the non-self-sustained oscillators affects the free running period under constant darkness and the ability to entrain to the light-dark cycle. We find that the proportion does not affect the free running period, but plays a significant role in the range of entrainment. We also find that its effect on the entrainment range depends on the region where the non-self-sustained oscillators are located. If the non-self-sustained oscillatory neurons are situated in the light-sensitive subregion, the entrainment range narrows when the proportion increases. If they are situated in the light-insensitive subregion, however, the entrainment range broadens with the increase of the proportion. We suggest that the heterogeneity within the light-sensitive and light-insensitive subregions of the SCN has important consequences for how the clock works.

All living things on the earth have evolved to enable synchronization of their endogenous rhythms to the external light-dark cycle. In mammals, a master clock regulating the circadian rhythms of behavioral and physiological activities is located in the suprachiasmatic nucleus (SCN) above the optic chiasm^1^,^2^,^3^,^4^. The SCN has two main functions. One is to maintain a free running rhythm under constant darkness, and the other is to synchronize the bodily rhythms to the external cycle of light and darkness^1^,^2^,^3^,^4^. Two parameters characterize these two functions, i.e. the free running period under constant darkness and the entrainment range to the external cycle^5^,^6^,^7^,^8^,^9^,^10^,^11^,^12^. The free running period and the entrainment range are around but not exactly 24 h, and are distinct among species. For example, for *Arivicanthis niloticus*, *Homo sapiens* and *Rattus morvegicus*, the free running periods are 23.8 h, 24.2 h and 24.4 h, and the entrainment ranges are from 22.5 to 25.5 h, from 20.5 to 29.0 h, and from 23.5 to 28.5 h, respectively^3^,^5^.

The endogenous rhythms of the SCN originate from self-sustained oscillations of the individual SCN neurons^13^,^14^, which are induced by a negative transcriptional feedback loop^4^,^15^. There are about twenty thousand neurons in the SCN, which can be distinguished into two subgroups, a light sensitive ventro-lateral (VL) part consisting of about 25% SCN neurons and light-insensitive dorso-medial (DM) part containing the remaining 75% SCN neurons^16^,^17^,^18^. The SCN neurons are coupled through neurotransmitters to form a network^19^,^20^, which are distinct in different regions: vasoactive intestinal polypeptide (VIP) is produced in the VL, arginine vasopressin (AVP) is secreted in the DM and gamma-amino butyric acid (GABA) operates between the VL and the DM^20^,^21^,^22^.

In addition to self-sustained oscillatory neurons, other phenotypes of SCN neurons such as neurons that show damped oscillations and arrhythmic neurons have also been observed^23^,^24^,^25^,^26^,^27^,^28^. Previously, it was suggested that there is a specialized or anatomically localized region for the rhythmic or arrythmic neurons. In particular, the DM neurons show endogenous rhythms, but the VL neurons are either damped or arrythmic^24^,^25^. However, recent experiments showed different findings. It has been found that the oscillatory phenotypes are not bound to specific regions, in other words, self-sustained oscillatory neurons, neurons that have damped oscillations and arrhythmic neurons are all observed in both the VL and the DM^26^. Furthermore, it has also been found that only a minority of SCN neurons can maintain self-sustained oscillations^26^. More recently, ref. 27 concluded that it is not easy to determine whether a neuron is a self-sustained oscillator or not, because noise can drive a non-self-sustained oscillator to be like a self-sustained oscillator. Hence, so far, the proportion of the SCN neurons for each oscillatory phenotype has not been obtained.

The non-self-sustained oscillators (e.g. damped neurons and arrhythmic neurons) have been found to play a role in the synchronization between neurons and the entrainment ability to the external cycle. The coupled non-self-sustained oscillators exhibit robust synchronization and are much more easily entrained to external cycle^28^,^29^,^30^. Yet, the impacts of the non-self-sustained oscillators in different regions have not been established. In the present study, we investigate the effects of the proportion of non-self-sustained oscillatory neurons to the whole neuronal population of the SCN on the two main functions of the SCN, being the free running period under constant darkness and entrainment ability to the external cycle. We also investigate the effects of this proportion in the different subgroups of neurons, being the light-sensitive VL subgroup and the light-insensitive DM subgroup. The Poincaré model is used to describe the SCN network, where the non-self-sustained oscillators are represented by the neurons that have zero intrinsic amplitudes.

## Methods

The Poincaré model is often used to mimic the SCN neuronal oscillators. There are two variables *x* and *y* for each neuronal oscillator^9^,^11^,^12^,^31^. The oscillators are coupled through a mean field *F*, and form an all-to-all network. The SCN composed of *N* oscillators is modelled as:


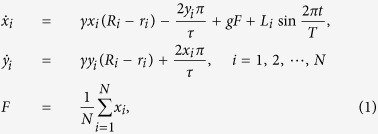


where *i* denotes the *i*_*th*_ neuronal oscillator. The parameters *γ*, *R*_*i*_, *r*_*i*_, *τ*, *g*, *L*_*i*_ and *T* represent the relaxation parameter, intrinsic neuronal amplitude, neuronal amplitude, intrinsic neuronal period, coupling strength, light sensitivity strength and the period of the external cycle respectively. The intrinsic neuronal amplitude parameter *R*_*i*_, is given a value of 0 for non-self-sustained oscillators and 1 for the self-sustained oscillators. Under constant darkness, there is no difference between the VL and the DM in the present model. To the external light-dark cycle, the difference between the VL and the DM is that the VL neurons receive the light signal and relay this light information to the DM neurons. We defined the light sensitive strength as *L*_*i*_ = *K*_*f*_ for *i* = 1, 2, …, *N*_*VL*_ and *L*_*i*_ = 0 for *i* = *N*_*VL*_ + 1, *N*_*VL*_ + 2, …, *N*, where *K*_*f*_ is the light intensity and *N*_*VL*_ is the number of neurons in the VL part of the SCN. For comparison, we also considered the case that all the SCN neurons are sensitive to an external signal such as temperature, i.e. *L*_*i*_ = *K*_*f*_ for *i* = 1, 2, …, *N*. The other parameters are chosen as in ref. 9: *γ* = 0.1, *τ* = 24, *g* = 0.1, and the amplitude is 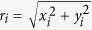.

The entrainment ability of the SCN to the external cycle can be represented by the lower limit of entrainment (*LLE*)^9^. The *LLE* is defined as the shortest *T* cycle that the SCN is able to synchronize to, and represents the entrainment range of the SCN. If the value of the *LLE* is smaller, the entrainment range is broader, and vice versa, if the value of the *LLE* is larger, the entrainment range is narrower. The synchronization or entrainment of the SCN to the external *T* cycle is determined by the difference between the periods of all SCN neuronal oscillators *T*_*i*_ and *T*. If 
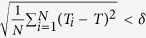
, where the difference *δ* is selected as 0.00001 h here, the SCN is assumed to be synchronized or entrained to the *T* cycle. The period of each neuronal oscillator *T*_*i*_ is calculated based on the evolution of *x*_*i*_. Key parameters are the proportions *p*, *p*_*VL*_ and *p*_*DM*_ representing the number of non-self-sustained oscillatory neurons in the SCN, VL and DM (where *R* = 0), respectively, to the total number of neurons in the SCN, VL and DM (*N*, *N*_*VL*_ or *N*_*DM*_), where the range of *p*, *p*_*VL*_ or *p*_*DM*_ is from 0 to 1. Note that in the present work, the non-self-sustained oscillatory neurons (with *R* = 0) in only one region of the SCN were studied, i.e. we did not investigate the neurons that have intrinsic amplitudes of zero in both the VL and the DM.

We used the fourth-order Runge-Kutta method for numerical simulations with time increments of 0.01 h. The initial 2,000,000 time steps (20,000 h) were neglected in order to avoid the effect of transients. The number of neurons *N* was set to 40 for the numerical simulations. The initial conditions for each variable were selected randomly from a uniform distribution in the range [0–1] for *x*_*i*_, *y*_*i*_. We also chose the case of *N* = 1000 and nonidentical neuronal periods *τ*_*i*_, and found that the results were in accordance with the results of *N* = 40.

## Results

### Simulation results

**Figure 1:**
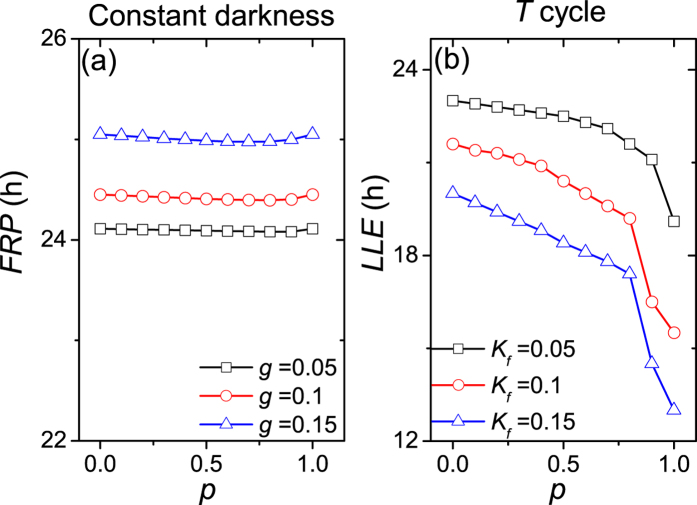
The dependence of the free running period *τ* under constant darkness (**a**) and the lower limit of entrainment (*LLE*) to the *T*-cycle (**b**) on the proportion *p* when all the SCN neuronal oscillators are sensitive to the external signal. The light intensity is *K*_*f*_ = 0 in (**a**), and the coupling strength is *g* = 0.1 in (**b**).

We examined the effects of the proportion *p* when all the SCN neurons are sensitive to the external signal, and when only the VL neurons are sensitive to the external signal. Figure 1 shows the influence of the proportion *p* on the free running period *FRP* and the lower limit of entrainment *LLE*, when all the SCN neurons are sensitive to the external signal. In (a), with the increase of the proportion *p*, the alternation of *FRP* is very small for each coupling strength *g*, in other words, the proportion *p* does not apparently affect *FRP*. In (b), the *LLE* of the SCN to an external cycle decreases with the increase of *p* for each light intensity, i.e. the entrainment range becomes broader with the increase of the proportion *p*.

**Figure 2:**
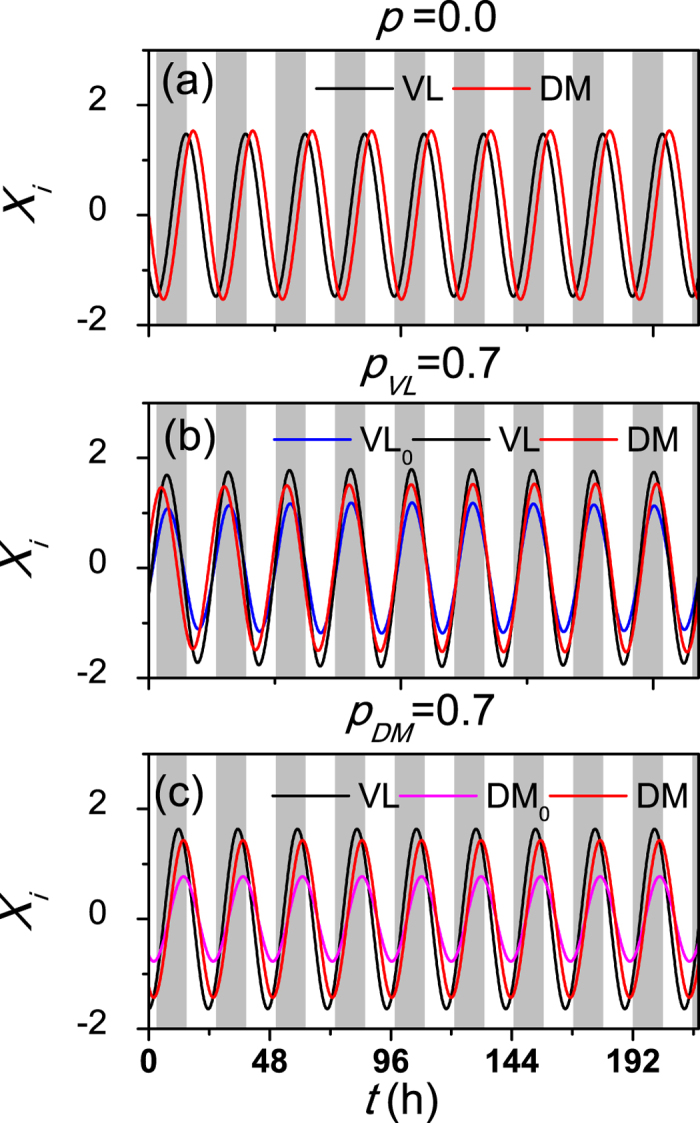
The evolutions in time of neuronal oscillators under *T*-cycle of 23.6 *h*, when the intrinsic amplitudes *R* in all SCN neurons are larger than 0 (**a**), or in 70% of VL neurons The amplitudes *R* are equal to 0 (*P*_*VL*_ = 0.7) (**b**) and in 70% of DM neurons the amplitudes *R* are equal to 0 (*P*_*DM*_ = 0.7) (**c**). The grey region corresponds to darkness, and VL_0_ and DM_0_ represent the neurons where the amplitudes are *R* = 0 in the VL and the DM respectively. The coupling strength is *g* = 0.1 and the number of SCN neurons is *N* = 40.

When only the VL neurons are sensitive to the external signal, an illustrative example for the effects of the proportion *p* on the entrainment of the SCN under an external cycle of 23.6 h is shown in Fig. 2. Typical evolutions in time for individual cells are presented, when the intrinsic amplitudes *R* for all SCN neurons are larger than 0 (*p* > 0) (a), or when the amplitudes *R* are equal to 0 for 70% of the VL neurons (*p*_*VL*_ = 0.7) (b) and when the amplitudes *R* are equal to 0 for 70% of the DM neurons (*p*_*DM*_ = 0.7) (c). In (a) and (c), both the VL neurons and the DM neurons are synchronized to the external cycle because the phase differences between the external cycle and the neurons remain constant over the cycles, while in (b) both the VL neurons and the DM neurons are not entrained to the external cycle because the phase differences fluctuate over time. Hence, the non-self-sustained oscillatory neurons in the VL lead to the reduction of the entrainment ability of the SCN to the external *T* cycle.

**Figure 3:**
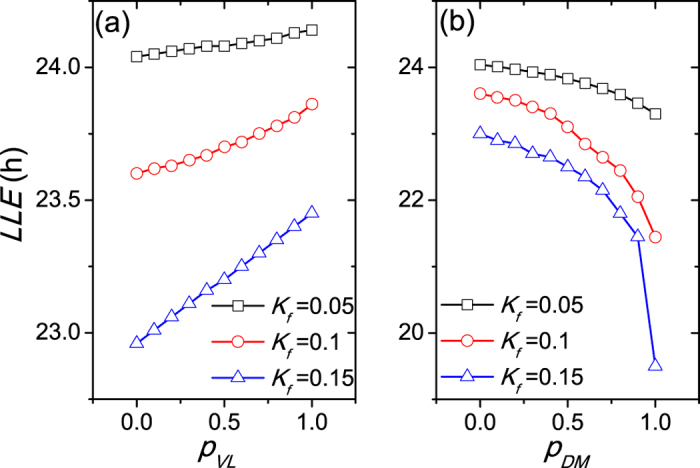
The dependence of the lower limit of entrainment (*LLE*) on the proportion *p* for the VL (**a**) and the DM (**b**). Three light intensities are taken into account, *K*_*f*_ = 0.05, 0.1 and 0.15. The coupling strength is *g* = 0.1 and the number of SCN neurons is *N* = 40.

We next quantify the relationship between the entrainment limit of the SCN and the proportion *p* of neurons that have an intrinsic amplitude of zero (*R* = 0) in the different regions (VL and DM) (Fig. 3). When the neurons with *R* = 0 are located in the VL, the *LLE* increases (and thus the entrainment range decreases) with the increase of the proportion *p*_*VL*_ for each light intensity in (a). On the contrary, when the neurons of *R* = 0 are located in the DM, the *LLE* decreases (and thus the entrainment range increases) with the increase of the proportion *p*_*DM*_ for each light intensity in (b). In both panels, the *LLE* is smaller, i.e., the entrainment range is broader, with larger light intensity. Thus, the proportion *p* of non-self-sustained oscillatory neurons in different SCN regions plays a distinct role in the entrainment range of the SCN.

The difference of the *LLE* between *p*_*VL*_ = 0 and *p*_*VL*_ = 1 is much smaller than it between *p*_*DM*_ = 0 and *p*_*DM*_ = 1 in Fig. 3, possibly because the VL accounts for about 25% SCN neurons and the remaining 75% SCN neurons are located in the DM. If *p*_*VL*_ = 1, only 25% of all SCN neurons are non-self-sustained, while if *p*_*DM*_ = 1, about 75% of all SCN neurons are non-self-sustained. As a result, the proportion of non-self-sustained oscillators in the VL plays a less prominent role, and the proportion of non-self-sustained oscillators in the DM predominates in the effects on the entrainment range.

**Figure 4:**
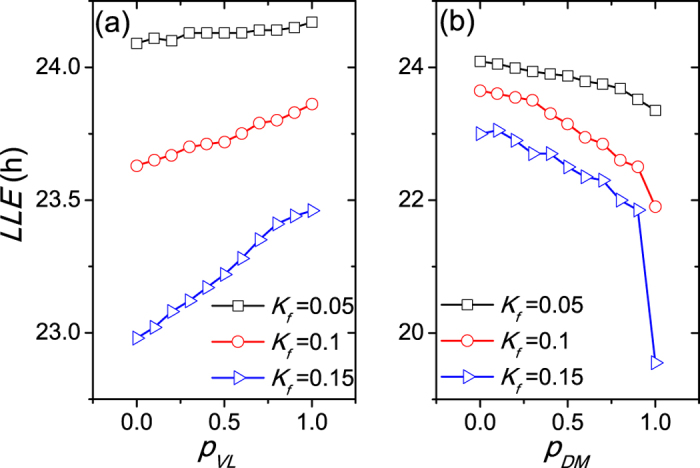
This figure corresponds to Fig. 3, but the intrinsic periods *τ*_*i*_ of the neurons are not identical and satisfy a normal distribution. The coupling strength is *g* = 0.1 and the number of SCN neurons is *N* = 1000.

**Figure 5:**
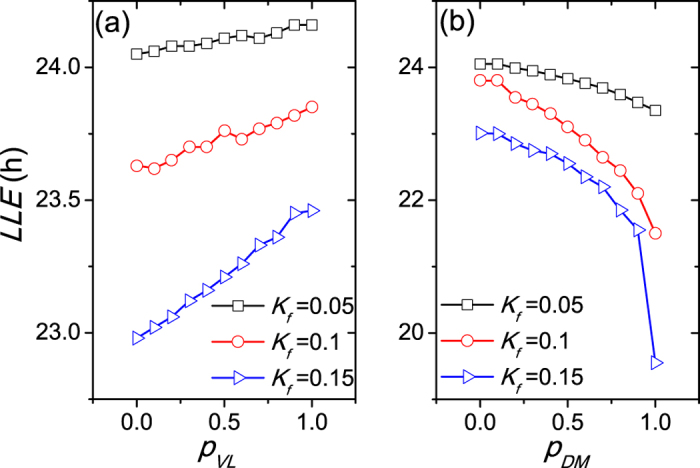
This figure corresponds to Fig. 3, but the coupling strength *g*_*i*_ are not identical and satisfy a normal distribution. The mean of the distribution is 0.1, the standard deviation of the distribution is 0.03*0.1 and the number of SCN neurons is *N* = 1000.

We also examined the effects of the proportion *p* when the intrinsic neuronal periods *τ*_*i*_ are nonidentical and satisfy a normal distribution with a mean equal to 24 h and a standard deviation 0.03*24 h (Fig. 4), and when the cellular coupling strengths *g*_*i*_ are nonidentical and satisfy a normal distribution with a mean equal to 0.1 and a standard deviation 0.03*0.1 h (Fig. 5). The results shown in Figs 4 and 5 are consistent with the previous results shown in Fig. 3.

### Analytical results

In order to explain that the proportions *p* of neurons with zero intrinsic amplitudes in different SCN regions play distinct roles in the *LLE* of the SCN, analytical results are given. For simplicity, the total number of the SCN neurons is assumed to be *N* = 2, i.e. one neuron *a* in the VL and the other neuron *b* in the DM. Eq. (1) is read as:


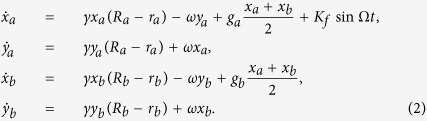


where 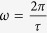, 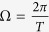, and other parameters are the same as in Eq. (1). For convenience, we transform Eq. (2) from Cartesian Coordinates to Polar Coordinates. Let *x*_*a*_ = *r*_*a*_ cos *θ*_*a*_, *y*_*a*_ = *r*_*a*_ sin *θ*_*a*_, *x*_*b*_ = *r*_*b*_ cos *θ*_*b*_, *y*_*b*_ = *r*_*b*_ cos *θ*_*b*_, *ϕ*_*a*_ = *θ*_*a*_ − Ω*t*, *ϕ*_*b*_ = *θ*_*b*_ − Ω*t*. Substituting them into Eq. (2), we obtain


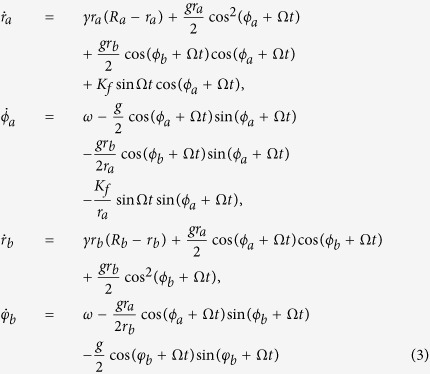


Considering the averaging method developed by Krylov and Bogoliubov as used in refs 9 and 32, *ϕ* has a lower time scale than Ω*t*. Letting *α* = 〈*ϕ*_*a*_〉 − 〈*ϕ*_*b*_〉, we get


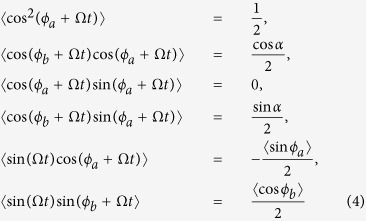


where 〈…〉 denotes the average in one light-dark cycle. For simplicity, we keep the non-averaged notation *r*_*a*_, *r*_*b*_, *ϕ*_*a*_ and *ϕ*_*b*_ in the following. When the SCN is entrained to the light-dark cycle, we have 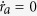, 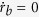, 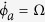, and 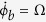. Substituting Eq. (4) into Eq. (3) we obtain Eq. (5):


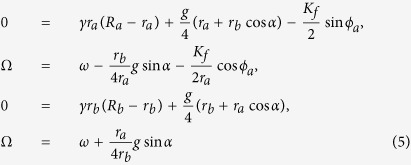


**Figure 6:**
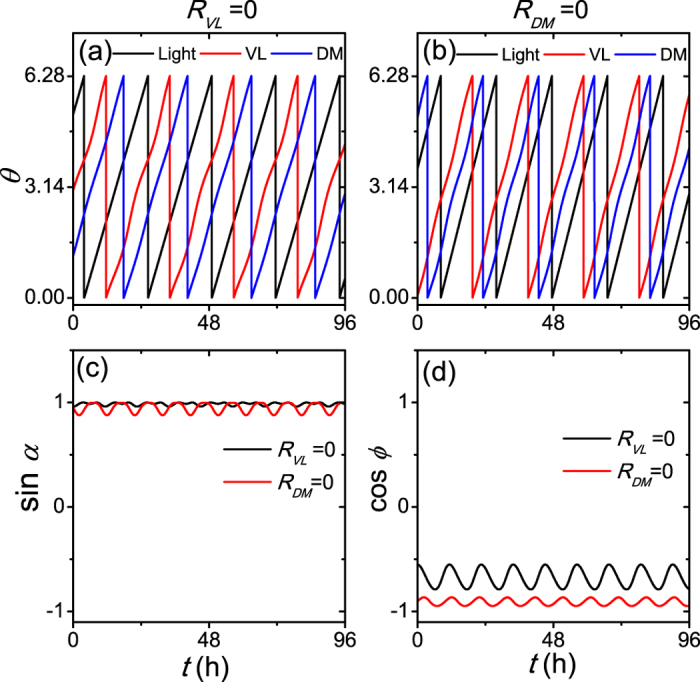
The phase information with the neuronal number *N* = 2 under the lower limit of entrainment. The evolutions of neuronal phases when the neuronal amplitude in the VL neuron is *R*_*VL*_ = 0 (**a**) and in the DM neuron is *R*_*DM*_ = 0 (**b**), the sine form of the phase difference *α* between the VL neuron and the DM neuron (**c**), and the cosine form of the phase difference *ϕ* between the VL neuron and the light cycle (**d**). The coupling strength is *g* = 0.1, and the light sensitivity is *K*_*f*_ = 0.15.

Intuitively, when Ω achieves the maximal value i.e. 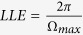, from the second equation and the last equation of Eq. (5), cos *ϕ*_*a*_ is assumed to be close to −1, and *sinα* ≈ z1. Shown in Fig. 6 confirms this intuition. Thus, Eq. (5) can be simplified as:


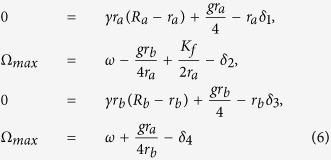


where *δ*_1,2,3,4_ are small terms. Consequently, we obtain:


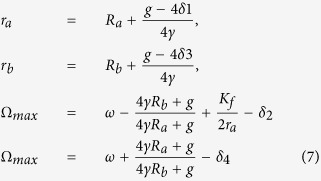


Visibly, from the last two equations of Eq. (7), the frequency Ω_*max*_ increases with the increase of *R*_*a*_ but with the decrease of *R*_*b*_. Consequently, if the value of *R*_*a*_ = 1 is decreased to *R*_*a*_ = 0, i.e, the value of *p*_*VL*_ = 0 is increased to *p*_*VL*_ = 1, the frequency Ω_*max*_ decreases. On the other hand, if the value of *R*_*b*_ = 1 is decreased to *R*_*b*_ = 0, i.e, the value of *p*_*DM*_ = 0 is increased to *p*_*DM*_ = 1, the frequency Ω_*max*_ increases. Hence, the theoretical analysis confirms our main finding that the proportion of the neurons with zero intrinsic amplitude in distinct locations play different roles in the entrainment range of the SCN.

## Discussion

In the last decades, the SCN neurons were thought to oscillate in a self-sustained manner^13^,^14^,^15^. This intrinsic neuronal oscillation drives the circadian rhythms of the SCN. However, it was found that some of the SCN neurons located in the VL are not self-sustained oscillators^24^,^25^. Recent experiments showed that, the non-self-sustained neuronal oscillators that either show damped oscillations or arrhythmic patterns are unrelated to specific regions of the SCN^26^. More recently, it was found that current experimental methods are insufficient to discriminate the self-sustained oscillators and the damped oscillators^27^. In most studies up to now, the VL neurons have been regarded as the non-self-sustained oscillators, and their effects on the synchronization between neurons and the entrainment ability of the SCN to external light-dark cycle have been examined by only looking at the VL neurons^29^,^30^.

Thus far, the effects of the non-self-sustained oscillatory neurons in different SCN regions are still unexplored. As a first step, based on the Poincaré model, we investigated the effects of the proportion of non-self-sustained oscillatory neurons on the two principal properties of the SCN, which are free running under constant darkness and the ability to entrain to the external cycle. Under the constant darkness, where there is no difference between the VL and the DM, the proportion does not affect the free running period. On the other hand, we found that the proportion of non-self-sustained oscillatory neurons does play a role in the entrainment range of the SCN to the external light-dark cycle. The role depends on the location of these neurons. If these neurons are located in the VL, the entrainment range narrows with the increase of the proportion of non-self-sustained oscillators. On the contrary, if these neurons are situated in the DM, the entrainment range broadens with the increase of this proportion. For comparison, if all the SCN neurons are sensitive to the external signal, the result is in accordance with the situation of these neurons being located in the DM.

One possible explanation for the distinct roles of the non-self-sustained oscillators in the different location is that, the non-self-sustained oscillators either in the VL or the DM may have smaller amplitudes than their self-sustained oscillatory counterparts and thus effectively reduce the mean field *F* which contributes to the coupling term in Eq. (1). The reduced mean field could lead to distinct effects on the entrainment ability of the SCN. If these non-self-sustained oscillators are located in the VL, where the neurons are sensitive to the light signals, the mean amplitude of the total VL neurons becomes smaller. As a result, the influence of the VL on the DM is reduced in that the entrainment ability of the SCN is decreased. If these non-self-sustained oscillators are located in the DM, where the neurons are light-insensitive and coupled to the VL neurons, the mean amplitude of the total DM neurons is smaller, thus the DM is more entrainable and the entrainment ability of the SCN is improved.

The opposite roles of the proportion of non-self-sustained oscillatory neurons in different regions suggest that the this heterogeneous network of the SCN is achieving higher flexibility to adjust to external influences than the homogeneous network. The different mechanisms in both regions should be investigated in an associationistic way, as investigating the factors in isolation may not apprehend the complete picture. With these findings we show that the heterogeneity within the subgroups of light-sensitive and light-insensitive subregions of the SCN has important consequences for how the clock works.

## Additional Information

**How to cite this article**: Gu, C. *et al.* The effects of non-self-sustained oscillators on the entrainment ability of the suprachiasmatic nucleus. *Sci. Rep.* **6**, 37661; doi: 10.1038/srep37661 (2016).

**Publisher’s note**: Springer Nature remains neutral with regard to jurisdictional claims in published maps and institutional affiliations.
